# Indications of Keratoplasty and Outcomes of Deep Anterior Lamellar Keratoplasty Compared to Penetrating Keratoplasty

**DOI:** 10.7759/cureus.13825

**Published:** 2021-03-11

**Authors:** Ahmed M Abdelaal, Albaraa H Alqassimi, Mohammad Malak, Hassan T Hijazi, Manal Hadrawi, Muhammad A Khan

**Affiliations:** 1 Surgery/Ophthalmology, King Saud Bin Abdulaziz University for Health Sciences/King Abdulaziz Medical City - Ministry of National Guard Health Affairs, Jeddah, SAU; 2 College of Medicine, Alfaisal University, Riyadh, SAU; 3 Department of Ophthalmology/Cornea and Anterior Segment Consultant, King Fahad Armed Forces Hospital, Jeddah, SAU; 4 Department of Ophthalmology/Ophthalmologist, King Fahad Armed Forces Hospital, Jeddah, SAU; 5 Department of Ophthalmology/Pediatric Ophthalmology and Strabismology Consultant, King Fahad Armed Forces Hospital, Jeddah, SAU; 6 Medical Education, King Saud Bin Abdulaziz University for Health Sciences, Jeddah, SAU

**Keywords:** deep anterior lamellar keratoplasty, keratoconus, keratoplasty, penetrating keratoplasty, corneal transplantation

## Abstract

Background

Corneal diseases are a significant cause of visual impairment and blindness. Despite the treatable nature of many corneal diseases before visual demise, many cases of advanced disease necessitating keratoplasty for visual rehabilitation are encountered. A mismatch between the number of corneal donors and potential recipients also exists worldwide, with underutilization of certain types of keratoplasty techniques that may allow more efficient use of this limited resource.

Methodology

A retrospective cohort study of all cases of optical corneal transplantations performed from January 1, 2015 to October 31, 2020 was performed. Indications for keratoplasty, type of keratoplasty, complications, intraocular pressure elevation, and best corrected visual acuity (BCVA) by category and range at different time intervals were collected and analyzed. Findings were compared between penetrating keratoplasty (PK) and deep anterior lamellar keratoplasty (DALK) for all indications, specifically for keratoconus (KCN).

Results

A total of 58 corneal transplants meeting our criteria were performed during the study period. PK was performed for 29 eyes, DALK for 28 eyes, and endothelial keratoplasty for one eye. The most frequently encountered indication was KCN (62.1%). The number of eyes with BCVA of 20/100 or better increased from preoperative BCVA, 37/58 eyes had BCVA worse than 20/100 before keratoplasty (63.8%), while at the time of last follow-up 45/58 eyes had BCVA of 20/100 or better (77.6%). At the time of last follow-up 16/58 had BCVA in the range of 20/20 to 20/40 (27.6%) and 29/58 eyes had BCVA in the range of 20/50 to 20/100 (50%). Comparison of all cases of PK to DALK for all indications showed significantly better BCVA by category at one year, at last follow-up, and BCVA range at last follow-up (p = 0.032, 0.001, and 0.014, respectively). Although better visual acuity results by category and range at one year and last follow-up, respectively, were observed in more patients undergoing DALK than PK, for KCN the results were not statistically significant (p = 1.00, 1.00, 0.417, and 0.374, respectively). Overall, 70% of recorded complications, intraocular pressure (IOP) elevations, and graft rejections were seen in eyes that underwent PK; however, these findings were not statistically significant (p = 0.297). Graft failures occurred more frequently with PK than deep anterior keratoplasty when analyzed for all indications of keratoplasty (p = 0.010).

Conclusions

Despite advancement and improvements in surgical techniques, statistics continue to show underutilization of the invaluable resource of donor corneas, with PK still being performed more than DALK for diseases that do not affect the endothelium. Our study found superior visual acuity outcomes of DALK as well as the advantages of less frequent complications, IOP elevations, graft rejections, and graft failures. We encourage ophthalmologists to utilize DALK in appropriate candidates to more fully utilize the scarce and potentially vision-restoring resource of donor corneal tissue.

## Introduction

Corneal diseases represent a major burden and are an important cause of visual disability and blindness [1]. Frequently, the main option for visual rehabilitation of patients with diseased corneas is corneal transplantation [2]. A unique characteristic that allows more corneal transplant procedures to be performed is the less stringent tissue matching requirements seen with other forms of transplantations, resulting in corneal transplantation being the most commonly performed tissue transplantation [3].

A large proportion of corneal blindness may be prevented with early access to ophthalmic care, identification, and timely intervention [4,5]. However, in the setting of delayed presentation, diagnosis, or treatment, the only remaining viable option for visual rehabilitation of eyes with corneal disease is corneal transplantation [2]. Due to the relative scarcity of quality corneal grafts for people who need them, corneal blindness remains an important prevalent global health issue [5]. This remains true despite rapid advancements and development of novel surgical instruments, techniques, and medications over the last few decades [6].

Great advancements in eye banking, corneal donor tissue preparation, and surgical techniques allow a single cornea to be used for multiple patients by utilizing a portion for anterior lamellar keratoplasty (ALK) in eyes with corneal diseases confined to the stroma and the remaining endothelium and descemet membrane portion for endothelial keratoplasty (EK) for eyes with isolated endothelial pathology. This has resulted in an increasing number of EK procedures performed; however, ALK has not experienced a similar surge [7]. The lack of popularity of ALK is true even for diseases confined to the corneal stroma such as keratoconus (KCN), post-refractive surgery ectasia, and scars following pterygium excision [8].

Indications for keratoplsty have also seen a change along with the advancements in medical care and socioeconomic standards in different parts of the world [9]. KCN, failed corneal transplants, congenital hereditary endothelial dystrophy, and corneal ulceration have become the most frequently encountered indications, while corneal degeneration, scarring, and pseudophakic bullous keratopathy (PBK) are encountered less frequently than they once were [9].

In this study, demographic data of patients undergoing any type of optical keratoplasty as well as the indications for keratoplasty are examined. Comparison between penetrating keratoplasty (PK) and deep anterior lamellar keratoplasty (DALK) for all indications, specifically for KCN, will be conducted to assess the occurrence of postoperative complications, graft rejection, graft failure, as well as best corrected visual acuity (BCVA) outcomes at different time intervals.

## Materials and methods

This is a retrospective cohort study including all cases of keratoplasty from January 1, 2015 until October 31, 2020 performed at the King Fahad Armed Forces Hospital in Jeddah, Saudi Arabia. All keratoplasties performed during the study period with the aim of improving vision (optical grafts) including patients with bilateral and/or repeat keratoplasty were included. Any keratoplasty performed outside our institute but followed in our clinics during the study period and keratoplasties performed in our institute for globe preservation (therapeutic and/or tectonic grafts) were excluded.

Demographic data, indication for keratoplasty, type of keratoplasty procedure, preoperative BCVA, postoperative BCVA at one year and last follow-up, postoperative intraocular pressure (IOP) elevation, postoperative complications, graft rejections, and graft failure were collected from the recipient’s medical records and recorded into spreadsheets.

BCVA for all recipient eyes was broadly categorized as better than or equal to 20/100 and worse than 20/100, as well as into smaller ranges as follows: 20/20 to 20/40, 20/50 to 20/100, 20/200 to 20/400, counting fingers, hand motion, light perception, and no light perception. BCVA was recorded at the following determined time intervals: preoperative BCVA, BCVA at one year postoperatively, and BCVA at the last follow-up postoperatively.

For eyes that developed IOP elevations following keratoplasty, an IOP of more than 24 mmHg after two or more weeks of treatment with topical prednisolone acetate (1%) were labeled as steroid-induced glaucoma if their IOP was subsequently controlled after changing postoperative prednisolone acetate (1%) and discontinuing any medication for lowering of IOP. For patients whose IOP remained more than 24 mmHg despite successfully changing their topical immunosuppressive medication, or those with documented angle closure, glaucomatous optic nerve, or visual field changes, who required continued topical medication treatment or surgical intervention for IOP control were labelled as secondary glaucoma following keratoplasty.

Statistical analysis

Data entry was conducted on Microsoft Excel program and statistical analysis was performed using SPSS version 24.0 (IBM Corp., Armonk, NY, USA). Qualitative variables were described as frequency and percentage, while quantitative variables were presented as median (interquartile range). For data comparison, chi-square test or Fisher exact test was used. The dependent variable in our analysis was type of current keratoplasty (PK and DALK) excluding the one EK performed during the study. Independent variables were postoperative complications, IOP elevation requiring intervention, graft rejection, graft failure, BCVA category, and range at one year following keratoplasty, as well as BCVA category and range at the time of last follow-up. Throughout the analysis, a p-value of <0.05 was considered significant.

## Results

A total of 58 corneal transplants meeting our inclusion criteria were performed during the study period. The youngest and oldest patients undergoing a corneal transplant in our study were 17 and 87 years of age, respectively, with a mean age of 41.7 years and a median age of 34.4 years. A total of 33 eyes undergoing keratoplasty were performed on males and the remaining 25 keratoplasties were performed on females. Mean follow-up time after keratoplasty was 20.76 months (standard deviation [SD], ±14.33).

BCVA prior to keratoplasty was worse than 20/100 in 37/58 (63.8%) eyes. The most common indication for keratoplasty in our study was KCN representing 36/58 (62.1%) of the overall corneal transplantations performed. A total of 29 eyes underwent PK, 28 eyes underwent DALK, and one eye underwent an EK. Table 1 summarizes the preoperative BCVA categories, BCVA ranges, keratoplasty indications, and type of keratoplasty performed.

**Table 1:** BCVA category and range before keratoplasty, indications for keratoplasty, and type of keratoplasty. BCVA = best corrected visual acuity; PBK = pseudophakic bulluos keratopathy; PK = penetrating keratoplasty; DALK = deep anterior lamellar keratoplasty; EK = endothelial keratoplasty

	Number (58)	Percentage
BCVA category before keratoplasty		
20/100 or better	21	36.2
Worse than 20/100	37	63.8
BCVA range before keratoplasty		
20/20–20/40	2	3.4
20/50–20/100	20	34.5
20/200–20/400	13	22.4
Counting fingers	19	32.8
Hand motion	3	5.2
Light perception	1	1.7
Indication for keratoplasty		
Keratoconus	36	62.1
PBK and corneal decompensation	11	19
Post-keratorefractive surgery ectasia	4	6.9
Herpetic keratitis	3	5.2
Failed grafts	2	3.4
Pellucid marginal degeneration	1	1.7
Epithelial downgrowth	1	1.7
Type of keratoplasty		
PK	29	50.0
DALK	28	48.3
EK	1	1.7

Results of best corrected visual acuity of all types of keratoplasty for all indications at one year and at last follow-up

BCVA at one year for the 43 eyes achieving that duration of follow-up was 20/100 or better in 31/43 eyes (72.1%) and worse than 20/100 in the remaining 12/43 (27.9%) eyes. BCVA at last follow-up for all 58 eyes was 20/100 or better in 45/58 (77.6%) eyes, with 16/58 (27.6%) eyes having a BCVA of 20/20 to 20/40 and 29/58 (50%) eyes having a BCVA of 20/50 to 20/100.

Of the 13/58 eyes which did not achieve a BCVA of 20/100 or better at last follow-up, 5/13 (38.5%) eyes were deemed to be as a consequence of refractive errors, with four being suture-related and one as a consequence of a change in refraction following cataract surgery with an intraocular lens in an eye with previous PK. Non-refractive causes of vision worse than 20/100 were seen in 7/13 (53.8%) eyes and occurred as a consequence of failed grafts with reduced graft clarity. One eye developed endophthalmitis during the first year after PK following microbial keratitis (MK) and consequently no light perception BCVA in later follow-up evaluations. One eye was deemed to have BCVA worse than 20/100 for both refractive and non-refractive influences due to suture-related refractive errors and diabetic macular edema. Table 2 shows the BCVA category and range at one year for the 43 eyes which achieved that duration of follow-up, as well as for all 58 eyes at their last follow-up visit.

**Table 2:** BCVA category and range following keratoplasty at one year and at last follow-up for all eyes. BCVA = best corrected visual acuity

	Number	Percentage
BCVA category one year after keratoplasty		
20/100 or better	31	53.4
Worse than 20/100	12	20.7
BCVA range one year after keratoplasty		
20/20–20/40	11	19.0
20/50–20/100	20	34.5
20/200–20/400	5	8.6
Counting fingers	4	6.9
Hand motion	2	3.4
Light perception	1	1.7
Total	43	100
BCVA category at last follow-up		
20/100 or better	45	77.6
Worse than 20/100	13	22.4
BCVA at last follow-up		
20/20–20/40	16	27.6
20/50–20/100	29	50.0
20/200–20/400	5	8.6
Counting fingers	2	3.4
Hand motion	4	6.9
Light perception	1	1.7
No light perception	1	1.7
Total	58	100
Reason for BCVA less than 20/100		
Refractive	5	38.5
Non-refractive	7	53.8
Combined	1	7.7
Total	13	100

Results of penetrating keratoplasty versus deep anterior lamellar keratoplasty for all indications

When comparing the 57 eyes that underwent either PK (29) or DALK (28), complications in the first year following the procedure (excluding IOP-related complications discussed later) occurred in 10/57 eyes, with PK representing a larger proportion with 7/10 (70%) and DALK with 3/10 (30%) of complications. Complications included suture-related wound leaks (two PK eyes), MK (two PK eyes), herpes simplex virus (HSV) recurrence (two PK eyes), graft vascularization with scarring (three DALK eyes), and traumatic hyphema and anterior chamber intraocular lens dislocation (one PK eye). One of the two eyes that developed MK went on to develop endophthalmitis and no light perception vision. However, the observed difference in the occurrence of postoperative complications between both types of keratoplasty was not statistically significant (p = 0.297).

IOP elevations requiring medical or surgical intervention were encountered in 10/57 eyes that underwent either PK or DALK (17.5%). Seven of the 10 (70%) eyes developed documented IOP elevation more than two weeks after their keratoplasty procedure and required topical treatment with eventual normalization of their IOP following cessation of topical steroids and IOP-lowering medications, and were labelled as steroid-induced glaucoma. Five of the seven eyes with steroid-induced glaucoma underwent PK (71.4%) and 2/7 eyes underwent DALK (28.6%). The other 3/10 (30%) cases of elevated IOP following keratoplasty also had documented elevation of IOP that required medical or surgical treatment with evidence of glaucomatous optic nerve and visual field damage, all occurring exclusively in eyes that underwent PK and were labelled as secondary post-PK glaucoma (PPKG). For the purpose of our analysis, steroid-induced glaucoma and secondary PPKG were grouped together into a larger group. A higher proportion of IOP elevation requiring medical or surgical intervention was seen following PK; however, no statistical difference was observed between PK and DALK (p = 0.297).

Graft rejections occurred in 10/57 eyes following PK or DALK (17.5%), with one eye having two different episodes of graft rejection related to recurrent HSV keratitis before eventual graft failure. Seven of the graft rejections occurred in eyes with PK (70%) and three in eyes with DALK (30%), despite the higher frequency of occurrence of graft rejections following PK; however, no statistical significance in the occurrence of rejections between the two types of keratoplasty was noted (p = 0.297). Graft failure occurred in 7/57 (12.3%) eyes. All cases were secondary graft failures and all occurred in eyes that underwent PK (p = 0.010).

DALK showed significantly better BCVA than eyes that underwent PK. At one year, 31/43 eyes that underwent DALK or PK achieved a BCVA of 20/100 or better, of which 19/31 (61.3%) eyes underwent DALK and 12/31 (38.7%) underwent PK (p = 0.032). BCVA range of 20/20 to 20/40 at one year was achieved in 11 eyes, with 4/11 (36.4%) eyes having underwent PK and 7/11 (63.6%) DALK . BCVA range at one year of 20/50 to 20/100 was achieved in 20 eyes, with 8/20 (40%) eyes undergoing PK and 12/20 (60%) DALK. Despite the larger proportion of better BCVA ranges at one year in eyes with DALK as opposed to PK, the difference observed was not statistically significant (p = 0.098). At the time of last follow-up, 44/57 eyes achieved a BCVA of 20/100 or better. BCVA via our categorization showed a significant difference between DALK and PK at the time of last follow-up, with 27/44 (61.4%) eyes that underwent DALK and 17/44 (38.6%) eyes that underwent PK achieving a BCVA of 20/100 or better (p = 0.001). Analysis of BCVA by ranges at the time of last follow-up was notable for 16/58 (27.6%) eyes that achieved BCVA of 20/20 to 20/40, with 8/16 (50%) eyes in both the PK and DALK groups. BCVA ranges of 20/50 to 20/100 at the time of last follow-up was achieved in 28/58 (48.3%) eyes, with 9/28 (32.1%) eyes undergoing PK and 19/28 (67.9%) eyes undergoing DALK. The difference in BCVA ranges at the time of last follow-up was also significantly better for DALK compared to PK (p = 0.014). Table 3 shows the comparison of outcomes between PK and DALK.

**Table 3:** Outcomes of PK versus DALK for all indications of keratoplasty. PK = penetrating keratoplasty; DALK = deep anterior lamellar keratoplasty; IOP = intraocular pressure; BCVA = best corrected visual acuity *Chi-square test, **Fisher’s exact test

		Type of current keratoplasty					
		PK		DALK		Total	P-Value
		n (29)	%	n (28)	%		
Postoperative complications	Yes	7	70.0	3	30.0	10	0.297**
	No	22	46.8	25	53.2	47	
IOP elevation requiring intervention	Yes	7	70.0	3	30.0	10	0.297**
	No	22	46.8	25	53.2	47	
Graft rejection	Yes	7	70.0	3	30.0	10	0.297**
	No	22	46.8	25	53.2	47	
Graft failure	Yes	7	100.0	0	0.0	7	0.010**
	No	22	44.0	28	56.0	50	
BCVA category one year after keratoplasty	20/100 or better	12	38.7	19	61.3	31	0.032*
	Worse than 20/100	10	83.3	2	16.7	12	
	Not applicable	7	50.0	7	50.0	14	
BCVA range one year after keratoplasty	20/20–20/40	4	36.4	7	63.6	11	0.098**
	20/50–20/100	8	40.0	12	60.0	20	
	20/200–20/400	4	80.0	1	20.0	5	
	Counting fingers	4	100.0	0	0.0	4	
	Hand motion	2	100.0	0	0.0	2	
	Not applicable	7	46.7	8	53.3	15	
BCVA category at last follow-up	20/100 or better	17	38.6	27	61.4	44	0.001*
	Worse than 20/100	12	92.3	1	7.7	13	
BCVA range at last follow-up	20/20–20/40	8	50.0	8	50.0	16	0.014**
	20/50–20/100	9	32.1	19	67.9	28	
	20/200–20/400	4	80.0	1	20.0	5	
	Counting fingers	2	100.0	0	0.0	2	
	Hand motion	4	100.0	0	0.0	4	
	Light perception	1	100.0	0	0.0	1	
	No light perception	1	100.0	0	0.0	1	

Results of penetrating keratoplasty versus deep anterior lamellar keratoplasty for keratoconus

Comparison of the 15 and 21 eyes that underwent PK and DALK for KCN, respectively (a total of 36 eyes), showed a total of 26 patients who achieved BCVA 20/100 at one year. PK represented 11/26 (42.3%) and DALK represented 15/26 (57.7%) of the eyes, which was not statistically significant (p = 1.00). Analysis of BCVA ranges at one year between both groups was also insignificant (p = 1.000). At the time of last follow-up, there were 35 patients who achieved a BCVA of 20/100 or better, with 14/35 (40%) eyes that underwent PK and 21/35 (60%) that underwent DALK; however, no statistical significance was observed (p = 0.417). At the last follow-up visit for KCN eyes that underwent keratoplasty, BCVA in the range of 20/20 to 20/40 was seen in 14/36 (38.9%) eyes, with both PK and DALK accounting for 7/14 (50%) eyes; BCVA in the range of 20/60 to 20/100 was seen in 21/36 (58.3%) eyes, with 7/21 (33.3%) eyes having had PK and 14/21 (66.7%) eyes having had DALK. Differences in BCVA ranges at the last follow-up were not statistically significant (p = 0.374). Four of the 10 documented graft rejections in our study occurred in KCN patients. Two eyes occurred in both PK and DALK with no statistical significance (p = 1.000). No eyes that underwent PK or DALK for KCN developed graft failure. Table 4 shows the comparison of PK and DALK performed for KCN.

**Table 4:** Outcomes of PK vs DALK for keratoconus. PK = penetrating keratoplasty; DALK = deep anterior lamellar keratoplasty; BCVA = best corrected visual acuity

		Type of current keratoplasty					
		PK		DALK		Total	P-Value
		n (15)	%	n (21)	%		
BCVA category one year after keratoplasty	20/100 or better	11	42.3	15	57.7	26	1.000
	Worse than 20/100	0	0.0	1	100.0	1	
	Not applicable	4	44.4	5	55.6	9	
BCVA range one year after keratoplasty	20/20–20/40	4	44.4	5	55.6	9	1.000
	20/50–20/100	7	43.8	9	56.3	16	
	20/200–20/400	0	0.0	1	100.0	1	
	Not applicable	4	40.0	6	60.0	10	
BCVA category at last follow-up	20/100 or better	14	40.0	21	60.0	35	0.417
	Worse than 20/100	1	100.0	0	0.0	1	
BCVA range at last follow-up	20/20–20/40	7	50.0	7	50.0	14	0.374
	20/50–20/100	7	33.3	14	66.7	21	
	20/200–20/400	1	100.0	0	0.0	1	
Graft rejection	Yes	2	50.0	2	50.0	4	1.000
	No	13	40.6	19	59.4	32	
Graft failure	Yes	0	0.0	0	0.0		Not applicable
	No	15	41.7	21	58.3	36	

## Discussion

Drastic changes in the indications for corneal transplantation have occurred over the last decades, with KCN becoming one of the leading indications [9]. Our study demonstrates a continuation of that trend with 36/58 (62.1%) eyes undergoing transplantation for KCN. We believe this to be multifactorial with indications which previously accounted for a higher number of corneal transplants such as trachoma, HSV keratitis, and PBK being supplanted due to better access to more advanced diagnostic and therapeutic ophthalmic care. Our study was also conducted in an area with a relatively high prevalence of KCN in both pediatric and adult age groups [10,11]. We believe the prevalence of KCN reflected on the mean and median age of 41.7 and 34.4 years of age, respectively, for patients undergoing keratoplasty in our study.

The majority of studies comparing BCVA for DALK and PK demonstrate similar visual outcomes in both groups [12-18]. When superiority for either procedure was noted, PK usually yielded better BCVA results [19,20]. Rare reports found better BCVA following DALK compared to PK [21]. In our study, we found a larger and more significant proportion of eyes that achieved BCVA of 20/100 or better at one year and at last follow-up for all indications after DALK (p = 0.032 and 0.001). When evaluating BCVA by range at one year and at last follow-up, DALK had a larger proportion of eyes with BCVA range of 20/20 to 20/40 and 20/50 to 20/100; however, this was not statistically significant at one year (p = 0.098), with statistical significance evident at the time of last follow-up (p = 0.014).

In the setting of KCN, some studies have shown PK to yield superior BCVA outcome than DALK [22,23]. Comparison of PK and DALK for KCN in our study revealed similar BCVA outcomes for both procedures, as reported by some other studies [17,24]. Although we had a higher proportion of better BCVA in our DALK group, no statistical significance was observed between PK and DALK in terms of BCVA for KCN. We believe our observation of a higher proportion of better BCVA in KCN patients who underwent DALK to be multifactorial, with the influence of an easier postoperative course and less endothelial cell loss major contributors. In our study, the rate of graft rejection in PK and DALK in KCN was similar (two eyes each). No episodes of graft failure occurred in any KCN eyes compared to other indications, reflecting the generally accepted good prognosis of PK for KCN [25].

Different complications are known to occur following PK and DALK [12]. However, complications following PK tend to be more frequent and devastating both intraoperatively and postoperatively [12,24]. In our study, 10 eyes experienced non-IOP-related complications, although they occurred more frequently in the setting of PK than DALK; however, the difference did not show significance (p = 0.297). A known disadvantage of PK is more endothelial cell loss compared to DALK [12,14]. We believe this to be reflected in our study by the lack of graft failures occurring during our study following DALK.

Another potential disadvantage of PK not observed in DALK is an IOP elevation from baseline [14]. A blinding secondary PPKG is also uniquely attributed to PK, with a recent systematic review and meta-analysis showing the rate of PPKG to be between 12.1% and 22.5% [26]. Our study demonstrated a larger proportion of IOP elevation requiring medical or surgical intervention for IOP control in the PK group (70%), including secondary PPKG; however, the observed higher frequency in eyes that had undergone PK was statistically insignificant. In total, 10/58 (17.2%) eyes developed either steroid-induced or secondary PPKG. Three of the 29 eyes (10.3%) that underwent PK throughout our study developed secondary PPKG. We believe the difference in our study from the range in the systematic review and meta-analysis to be due to differences in definitions.

From our experience, DALK is an excellent procedure for eyes with stromal pathology and a non-diseased endothelium. Our comparison of PK and DALK for all indications as well as specifically for KCN, along with observations from our clinical practice, have shown favorable outcomes of DALK with many advantages over PK. A less aggressive postoperative steroid course, shorter time to graft stability, and suture removal along with the eliminated risk of endothelial graft rejection ease the burden of surgery for both patients and ophthalmologists [12]. However, DALK still seems to lack popularity among ophthalmologists even for diseases with no endothelial pathology [8]. We believe this to be due to the technical difficulty of DALK, steep learning curve, and intraoperative complications which may lead to conversion to PK.

Our study is not without limitations; despite the inclusion of all indications for keratoplasty in our study, KCN represented a significant majority, which may affect the generalizability of our results. We also accept that longer follow-up periods following keratoplasty would be more ideal to give our results a stronger meaning. However, due to less endothelial cell loss following DALK, we believe that longer follow-up may demonstrate superiority for eyes with DALK in terms of BCVA and graft survival.

## Conclusions

We strongly advocate that ophthalmologists train and familiarize themselves with the DALK procedure and believe the advantages, including fewer complications, relatively less rigorous postoperative steroids, less strenuous follow-up course, lack of endothelial rejection, along with similar if not superior visual outcomes for DALK, as encountered in our study, warrant DALK to be the procedure of choice for corneal pathology sparing recipient endothelium. This will also allow for the scarce resource of donor corneal tissue to more effectively serve a larger number of those in need, especially in areas reliant on international donors.
